# DNA-AP sites generation by Etoposide in whole blood cells

**DOI:** 10.1186/1471-2407-9-398

**Published:** 2009-11-16

**Authors:** Emilio Rojas, Patricia Mussali, Efrain Tovar, Mahara Valverde

**Affiliations:** 1 Departamento de Medicina Genómica y Toxicología Ambiental Instituto de Investigaciones Biomédicas. Universidad Nacional Autónoma de México D.F. C.P. 04510, México; 2 Centro de Educación Ambiental e Investigación, Sierra de Huautla (CEAMISH), UAEM. Av. Universidad No. 1001, Col. Chamilpa, Cuernavaca, Morelos, CP 62210, México

## Abstract

**Background:**

Etoposide is currently one of the most commonly used antitumor drugs. The mechanisms of action proposed for its antitumor activity are based mainly on its interaction with topoisomerase II. Etoposide effects in transformed cells have been described previously. The aim of the present study was to evaluate the genotoxic effects of this drug in non-transformed whole blood cells, such as occurs as collateral damage induced by some chemotherapies.

**Methods:**

To determine etoposide genotoxicity, we employed Comet assay in two alkaline versions. To evaluate single strand breaks and delay repair sites we use pH 12.3 conditions and pH >13 to evidence alkali labile sites. With the purpose to quantified apurinic or apyrimidine (AP) sites we employed a specific restriction enzyme. Etoposide effects were determined on whole blood cells cultured in absence or presence of phytohemagglutinin (PHA) treated during 2 and 24 hours of cultured.

**Results:**

Alkaline (pH > 13) single cell gel electrophoresis (SCGE) assay experiments revealed etoposide-induced increases in DNA damage in phytohemaglutinine (PHA)-stimulated blood and non-stimulated blood cells. When the assay was performed at a less alkaline pH, 12.3, we observed DNA damage in PHA-stimulated blood cells consistent with the existence of alkali labile sites (ALSs). In an effort to elucidate the molecular events underlying this result, we applied exonuclease III (Exo III) in conjunction with a SCGE assay, enabling detection of DNA-AP sites along the genome. More DNA AP-sites were revealed by Exo III and ALSs were recognized by the SCGE assay only in the non-stimulated blood cells treated with etoposide.

**Conclusion:**

Our results indicate that etoposide induces DNA damage specifically at DNA-AP sites in quiescent blood cells. This effect could be involved in the development of secondary malignancies associated with etoposide chemotherapy.

## Background

In the last decade, etoposide (also known as VP-16213) has been one of the most commonly used agents for treating a number of malignancies. Etoposide is a semi-synthetic derivative of epipodophyllotoxin derived from the plant *Podophyllum peltatum *[1-3]. Its primary intracellular target, topoisomerase II, alters DNA topology by passing an intact double helix through a transient double stranded break that it generates in a separate nucleic acid segment [4-6].

Topoisomerase II is required to resolve knots and tangles in the genetic material that are produced by physiological processes such as DNA recombination and replication [7-12]. In the absence of topoisomerase II, cells are unable to segregate daughter chromosomes and die of mitotic failure [13].

In contrast to most drugs that target specific enzymes, etoposide and other topoisomerase II-targeting anticancer agents act in a subtle manner. Rather than blocking the activity of this essential enzyme, etoposide kills cells by increasing the concentration of topoisomerase II-DNA cleavage complexes [7,12,14-16]. This action converts topoisomerase II into a potent cellular toxin that fragments the genome. Consequently, etoposide has been deemed a topoisomerase II poison, distinct from drugs that inhibit the overall catalytic activity of an enzyme [7,12,14-18]. It has been known for more than a decade that etoposide stabilizes topoisomerase II-associated DNA breaks, thereby abolishing the ability of the enzyme to ligate cleaved nucleic acid molecules [7,12,16,19-21]. Specifically when etoposide interacts with topoisomerase IIα, it traps the enzyme in a covalently bound form with its DNA substrate [5,22].

The topoisomeriase IIα-DNA complex is stabilized with the etoposide molecule by hydrogen bonds with the nucleic acid bases, and this stabilized complex thus prevents re-ligation of DNA by topoisomerase IIα [23,24]. Both double-and single-strand breaks (SSBs) in DNA can be produced by etoposide.

The production of free radicals during etoposide metabolism has also been observed [25-27]. An orthoquinone metabolite of etoposide can be transformed into a hydroquinone [21]. When oxidized, hydroquinones give rise to hydroxyl radicals, which may ultimately contribute to etoposide-associated SSBs in DNA [28]. Although, the etoposide mechanism of action is well described in transformed cells, is important to know the effects generated in non-transformed whole blood cells as they are also exposed to the antineoplastic drug.

The Single Cell Gel Electrophoresis (SCGE) assay, also known as the comet assay, has been proposed as a sensitive, reliable and rapid method for detecting DNA SSBs, alkali labile sites (ALSs), and delayed repair sites (DRSs) in eukaryotic cells under extremely alkaline conditions (pH > 13) [29,30]. Meanwhile, the SCGE assay reveals only SSBs and DRSs under less extreme alkali conditions (i.e. pH 12.3). Thus by comparing the SCGE results obtained at pH 12.3 to those obtained pH >13, it is possible to discriminate the accumulation of apurinic and apyrimidinic sites (AP sites), which produce ALSs, from other forms of DNA damage.

In this study, we used the alkaline SCGE assay at pH 12.3 and pH >13 in non-stimulated and PHA-stimulated human blood cells to assess the genotoxicity associated with etoposide-induced oxidative stress in non-transformed cells. We performed follow-up assays with exonuclease III enzyme (Exo III) to detect DNA-AP sites within the genome [31]. The effect of co-treatment with an anti-oxidant, on etoposide genotoxicity was also examined. If etoposide treatment generates the production of reactive oxygen species, principally phenoxyl radicals, in non-stimulated whole blood cells, then exposure to an antioxidant should reduce the extent of DNA damage induced.

## Methods

### Chemical and reagents

Normal agarose, low melting point agarose (LMPA), ethidium bromide, Tris, Na_2_EDTA, DMSO (dimethyl sulfoxide), Phytohemaglutinin (PHA), Triton X-100, RPMI-1640 medium, and etoposide were obtained from Sigma Chemical Co. (St. Louis, MO), NaOH and NaCl were obtained from Merck (Mexico) and Baxter (Mexico), respectively. Exo III was obtained from Amersham Life Science (Piscataway, NJ, USA). Ascorbic acid (AA), also known as vitamin C, was obtained from ICN (Mexico).

### Human blood cells and treatments

The protocol was approved by the Ethics committee of Instituto de Investigaciones Biomédicas at Universidad Nacional Autónoma de México. Whole blood samples were obtained by vein punction from normal healthy volunteers, who were non-smokers and not taking any medications. PHA-stimulated and non-stimulated whole blood cells were treated for 2 or 24 h with different etoposide concentrations (0, 2.07, 20.7 and 207 μM), in the presence of RPMI-1640 culture medium and maintained at 37°C under 5% CO2 conditions.

The etoposide concentrations were determined by assuming a body surface area of 1.63 m^2 ^for the volunteers and calculating a dose equivalent to that used clinically for hematological malignancies. The calculated equivalent of the clinical dose was taken as the highest concentration applied in our study.

PHA-stimulated whole blood was first incubated for 6 h at 37°C under 5% CO_2 _conditions in 1 ml of RPMI-1640 culture medium with 71 μl of PHA, and then treated with etoposide as described above. To test the attenuation of etoposide AP-site generation in non-stimulated blood cells, the cultures were treated at the same time with AA (200 μM).

### Viability

The dual cell-stain assay described by Hartman and Speit [32] was employed to determine the viability of the PHA-stimulated and the non stimulated whole blood cells after etoposide treatments. The analysis was performed with a fluorescence microscope (Olympus BX60); 4 fields and at least 400 cells per slide were scored. The results were expressed as percentage of cells alive relative to controls.

### Single cell gel electrophoresis

The alkaline comet assay was performed essentially as described previously [33]. Briefly, after the experimental treatment was applied, 20-μl samples of whole blood, both PHA-stimulated and non-stimulated, were dissolved in 0.5% LMPA, spread onto microscope slides precoated with 0.5% agarose, and covered with an additional 0.5% LMPA layer. The cells were then lysed in a high salt and detergent solution (2.5 M NaCl, 10 mM EDTA, 10 mM Tris pH 10, with fresh 10% DMSO and 1%Triton x-100), for at least 1 h at 4°C. Subsequently, the cells were placed in a horizontal electrophoresis chamber and exposed to an alkaline solution (300 mM NaOH, 1 mM Na_2_EDTA, pH >13) for 20 min to allow the DNA to unwind. For DNA electrophoresis, a 25-V electric current (300 mA, 0.8 V/cm) was applied for 20 min. All technical steps were conducted under very dim indirect light. After electrophoresis, the slides were gently removed and the alkaline pH was neutralized by application of 0.4 M Tris, pH 7.5. The slides were dehydrated in two steps with absolute ethanol for 5 min each. Ethidium bromide (75 μl of a 20 μg/ml solution) was added to each slide and a coverglass was placed on the gel.

We performed the comet assay at pH 12.3 as described in our previous report [33]. The comet assay was also used in combination with Exo III as described by Gedik et al. [34]. Briefly, cells were centrifuged at 200 × *g *for 3 min at 4°C, dispersed in 75 μl 1% low melting point agarose at 37°C, placed on a microscope slide an processed for the comet assay. For each sample, we divided the gel in two parts, the upper part of the gel was incubated with ExoIII and buffer and lower part with buffer alone. The parts were separated by a coverglass.

The measures of AP sites were obtained by subtraction of the mean comet assay score with enzyme buffer alone from that with ExoIII. DNA migration was analyzed on an Olympus BMX60 microscope with epifluorescence equipment (with a 515-560-nm excitation filter and a 590-nm barrier filter). DNA migration measurements (tail image length, in microns) were made with a scaled ocular. To identify the tail, the head of the comet was defined as the most brilliant circular region in the image. One hundred cells were scored for each treatment condition. All experiments were conducted in triplicate and scored in a double blind manner.

### Statistical analysis

All statistical analyses were performed with STATISTICA software version 5 from STAT Soft Inc. USA (1996). The Mann-Whitney U test was used to determine DNA damage statistical differences between control cells and those treated with etoposide. Student's t test was used to compare cell viability, and Exo III recognition sites between control and etoposide-treated cells. For the evaluation of ALS, we used the Shapiro-Wilk "W" test which is used to probe normality [35]. We compared each treatment with its particular control (2 h non-stimulated, 2 h stimulated, 24 h non-stimulated and 24 h stimulated) and with each etoposide concentration (0.0, 2.07, 20.7, 207.0). The results reported show that *W *test was not significant in all case (2 h non-stimulated, W = 0.867, P = 0.06148; 24 h non-stimulated, W = 0.9093, P = 0.20902); 2 h stimulated, W = 0.91198, P = 0.22618; 24 h stimulated, W = 0.93917, P = 0.48735, then the hypothesis that the respective distribution is normal was accepted. We performed a one way ANOVA test in order to detect if there was an effect of the etoposide concentration (0.0, 2.07, 20.7, 207.0) on the formation of akali-labile sites (ALS) in stimulated and non-stimulated cells at 2 h and 24 h.

Thereafter, a multiple comparison test (Tukey) was used to determine the significant differences between group means, particularly we analyzed if the control differed significantly with each etoposide concentration [35]. The relationship between ALS index and net enzyme recognition sites was analyzed by Pearson's correlation.

## Results

### Viability

Our observations of the cells following the dual cell-stain method for PHA-stimulated and non-stimulated whole blood cells revealed that cell viability was high after etoposide treatment for 2 h and 24 h. As shown in table 1, we observed cell viability rates that exceeded 70%.

**Table 1:** Viability of non-stimulated and PHA-stimulated blood cells treated with etoposide as determined by dual-cell stain method.

Etoposide	2 h treatment non-stimulated blood cells	2 h treatment PHA-stimulated blood cells
**0 μM**	100 ± 5.5	100 ± 5.6
**2.07 μM**	95.5 ± 8.5	88.6 ± 3.9
**20.7 μM**	99.2 ± 2.2	83.2 ± 8.2
**207 μM**	91.1 ± 8.2	75.6 ± 7.6

**Etoposide**	**24 h treatment non-stimulated blood cells**	**24 h treatment PHA-stimulated blood cells**

**0 μM**	100 ± 2.3	100 ± 3.9
**2.07 μM**	100 ± 3.7	96.7 ± 3.4
**20.7 μM**	100 ± 8.0	96.7 ± 3.4
**207 μM**	93.8 ± 8.2	81.4 ± 9.9

Viability % ± SD. Each cell represents the average of three independent experiments.

### Single cell gel electrophoresis

**Figure 1:**
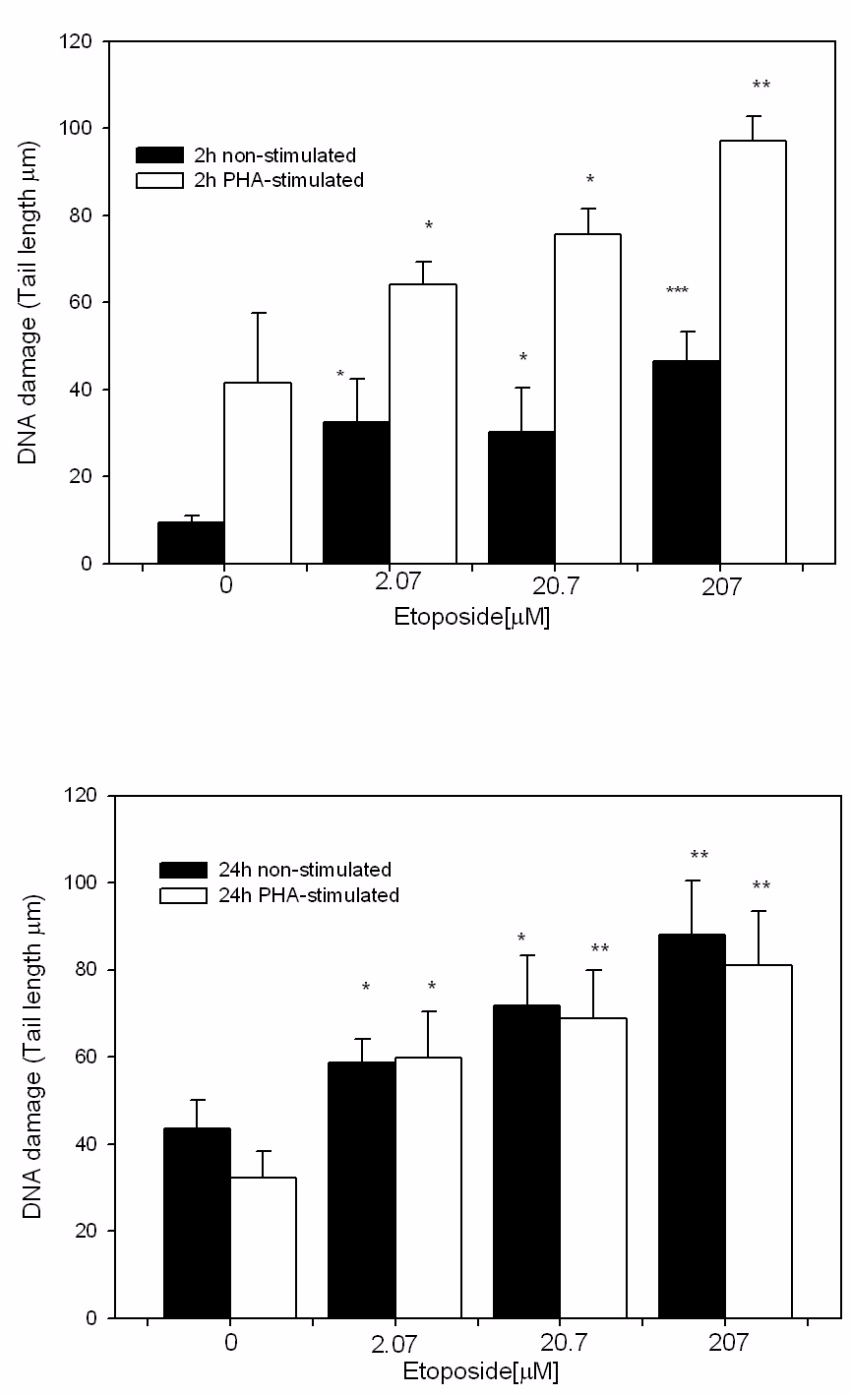
**DNA damage in non-stimulated and PHA-stimulated blood cells caused by 2-h and 24-h etoposide treatment at the indicated doses detected by Comet Assay at pH > 13**. A) 2-h treatment; B) 24-h treatment. Each bar represents the mean value of three independent experiments. Data were analyzed using the Mann-Whitney U test. (*= p < 0.05, ** = p < 0.01, *** = p < 0.001 vs. control).

**Figure 2:**
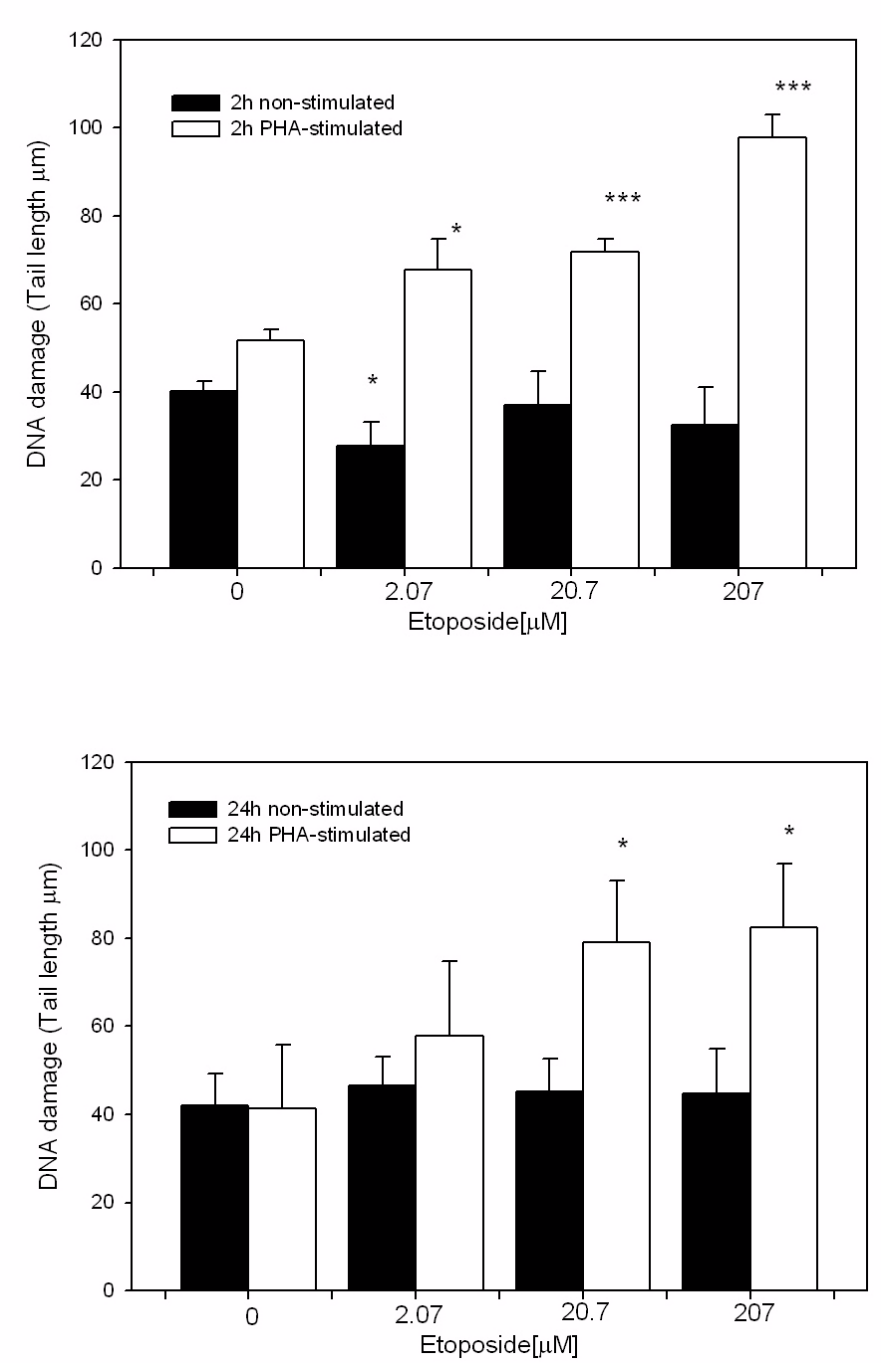
**DNA damage in non-stimulated and PHA-stimulated blood cells caused by 2-h and 24-h etoposide treatment at the indicated doses detected by Comet Assay at pH = 12.3**. A) 2-h treatment; B) 24-h treatment. Each bar represents the mean value of three independent experiments. Data were analyzed using the Mann-Whitney U test. (*= p < 0.05, *** = p < 0.001 vs. control).

SCGE at pH >13, which reveals SSBs, DRSs and ALSs, revealed a dose-dependent effect of etoposide treatment (2.07, 20.7 and 207 μM) on cells treated for 2 h and 24 h (Figure 1A, B respectively). We observed an increase in DNA-damage in all etoposide treatments showing the highest effect at 207 μM with respect to the control. However, SCGE assay performed at pH 12.3, which detect SSBs and DRSs, but not ALSs, showed genotoxic effects in the PHA-stimulated whole blood cells only at both treatment durations (Figure 2). These results demonstrate oxidative DNA damage generated by etoposide in non-stimulated blood cells, among others sources of ALS generation [35].

### Alkali labile site (ALS) index

**Figure 3:**
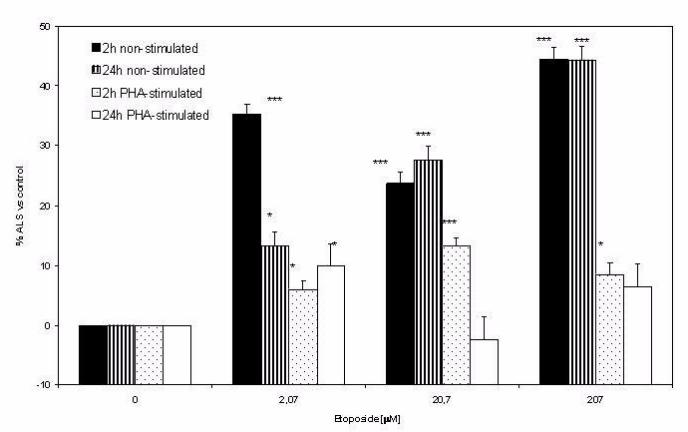
**Percentage of ALS relative to controls after 2-h and 24-h of etoposide treatment for both non-stimulated and PHA stimulated blood cells**. Data were analyzed using one way Anova test. (* p < 0.05 vs. control; *** p < 0.001 vs. control).

The ALS index was determined as the difference between the DNA damage detected at pH 12.3 and the damage estimated at pH>13 by the SCGE assay. All data were normalized with respect to the controls. As shown in Figure 3, non-stimulated whole blood cells had higher ALS index values than PHA-stimulated cells treated with etoposide. Control cells differed from etoposide-treated cells in all conditions except in PHA-stimulated cells treated for 24 h. The ANOVA analysis showed that there was a significant effect of etoposide concentration (0.0, 2.07, 20.7, 207.0) on the formation of ALS in all treatments: 2 h non-stimulated (F_3 _= 442.97, P < 0.0000), 24 h non-stimulated (F_3 _= 246.66, P < 0.0000) 2 h stimulated (F_3 _= 50.35,> P < 0000) 24 h stimulated (F_3 _= 9.52, P < 0.01).; these findings were consistent with the SCGE data (Figure 3).

### DNA AP-sites detection

**Figure 4:**
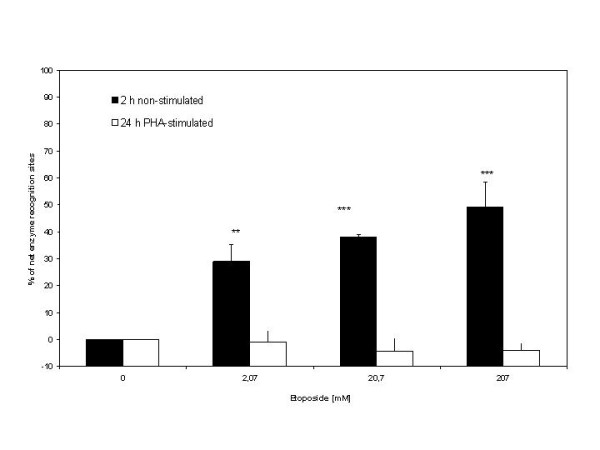
**Percentage of DNA-AP sites relative to controls evidenced by Exo III in non-stimulated blood cells treated for 2-h and PHA-stimulated blood cells treated 24-h with etoposide**. Data were analyzed using one way Anova test. (**p < 0.01 vs. control; ***p < 0.001 vs. control).

To test the hypothesis that ALSs generated by etoposide-oxidative stress could develop into DNA-AP sites, we used the enzyme Exo III, which recognizes this kind of DNA lesions. This analysis was performed in non-stimulated whole blood cells treated for 2 h with etoposide, a condition which produces a high rate of ALS induction, and the results were compared with the data from the PHA-stimulated whole blood cells exposed to etoposide for 24 h. As shown in figure 4, we observed a relationship between percentage of AP-sites and etoposide concentration in non-stimulated whole blood cells relative to PHA-stimulated blood cells subjected to the longer treatment.

**Figure 5:**
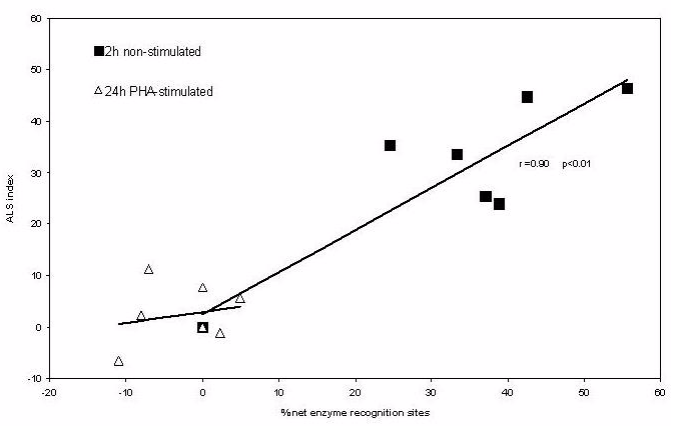
**Pearson's correlational analysis between the percent of DNA-AP sites as evidenced by Exo III and ALS index in non-stimulated blood cells treated for 2 h and PHA-stimulated blood cells treated for 24 h with etoposide**.

Because the presence of ALSs in non-stimulated whole blood cells was inferred indirectly by comparing the SCGE assays under the two pH conditions, it was important to test whether these putative ALSs involved AP-sites. Therefore we examined whether there was an association between the ALS index data and the DNA AP-sites data, as revealed by the use of Exo III. Indeed, we found a significant positive correlation (r = 0.90; p < 0.01) between ALS index value and the percent of DNA-AP sites detected by Exo-III (Figure 5).

**Figure 6:**
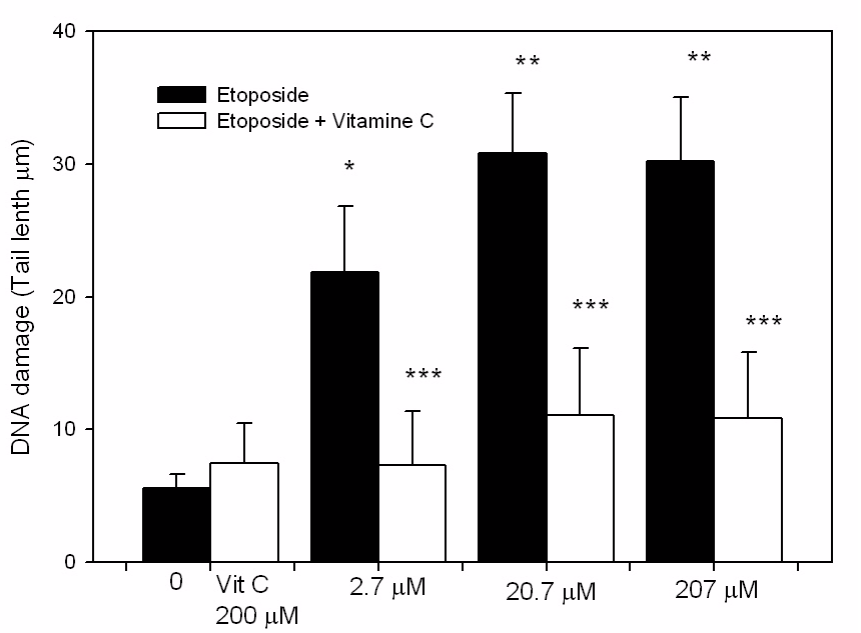
**DNA damage induced by etoposide (0, 2.07, 20.7 or 207 μM) in non-stimulated blood cells treated for 2-h (open bars) and DNA damage inhibition produced by AA in non-stimulated blood cells treated for 2 h with etoposide (solid bars)**. Every bar represents the mean value of three independent experiments. Data were analyzed using the Mann-Whitney U test. (*= p < 0.05; **= p < 0.005 vs. control; ***= p < 0.001 vs. corresponding no AA condition).

If etoposide treatment induces ROS, principally phenoxyl radicals in non-stimulated whole blood cells, then an antioxidant exposure should reduce the DNA damage induced by etoposide. As shown in figure 6, we found that a 2-h treatment course with the antioxidant AA (200 μM) concurrent with the 2-h etoposide treatment reduced the DNA damage induced by etoposide in non-stimulated whole blood cells. This finding indicates that AA provided some level of protection for the non-stimulated blood cell DNA in the oxidative micro-environment generated by the etoposide.

## Discussion

Etoposide affects chromatin function by directly and physically interfering with topoisomerase IIα enzyme activity. Topoisomerase IIα is considered to be an important player in the maintenance of the DNA double helix, due to its capacity to regulate conformational changes in DNA, in normal processes such as replication, transcription or condensation and segregation of chromosomes [4-6].

Topoisomerase IIα activity fluctuates with the cell cycle; its levels elevate as the cells progress through the cycle toward mitosis [36-39]. Thus as structural maintenance of DNA is most challenged during DNA replication, it is expected that cycling cells would be the most susceptible to damage in the presence of etoposide. Because non-stimulated cells are not cycling, they have relatively low Topoisomerase II activity compared to PHA-stimulated whole blood cells, and thus would be expected to be relatively insensitive to DNA-damaging effects of etoposide.

The present findings of etoposide-induced DNA damage in non-stimulated cells differ from the findings of Olive and Banath [40] which indicated an absence of DNA damage induction by etoposide in non-cycling cells. This discrepancy is most likely due to inherent differences between the cells used in the experiments. Olive and Banath used colon carcinoma cells (WiDr), while we used human lymphocytes from healthy donors. Lymphocytes, such as those used here, are normally arrested in the G0 phase and non-transformed.

To asses the molecular processes involved in the etoposide treatment-induced breaks, we compared the non-stimulated whole blood cells genotoxicity data generated by the SCGE assay under both highly basic (= 12.3) and extremely basic (> 13) pH conditions and thus generated the ALS index values (see results section). This comparison enables the presence of ALSs to be deduced indirectly because the oxidative response capable of generating apurinic or apyrimidic sites (AP sites) is pH-dependent [41,42]. The ALS index data indicated that ALSs, which can be generated by an oxidative stress [6,33], constituted the primary form of DNA damage induced by etoposide in whole blood cells.

To asses whether oxidative stress was responsible for the DNA damage observed in the etoposide-treated non-stimulated cells, we used the SCGE assay in combination with Exo III, an enzyme that recognizes AP sites in DNA. The results corroborated the presence of DNA-AP sites in the genomes of the non-stimulated cells treated with etoposide. Moreover, when non-stimulated cells were exposed to etoposide in the presence of the antioxidant AA, less DNA damage was observed. Thus the ability of the antioxidant to protect the DNA suggests that phenoxyl radicals are the major radicals involved in the oxidative DNA-damage induced by etoposide, especially in non-stimulated cells [[27,43], and [44]].

Interestingly, our results suggest that the molecular events by which DNA breaks are generated in PHA-stimulated and non-stimulated cells are quite different. The damage observed in the PHA-stimulated whole blood cells could be explained by the classical events ascribed to topoisomerase II poisons [12].

These have two principal components: DNA strand breaks due to the inhibition of topoisomerase II by etoposide and etoposide-quinone free radical effects [25,45]. Tornov and colleagues [46] observed DNA damage in leukocytes evidenced by SCGE assay at pH 13, suggesting that etoposide might cause oxidative damage in leukocytes by a mechanism involving inhibition of the enzyme topoisomerase IIβ. Although the exact function of this enzyme has not been resolved, it is known that its concentration is generally independent of cell cycle and cell growth [[12,37,39], and [46]]. In addition, topoisomerase IIβ is expressed at a higher level than topoisomerase IIα in human peripheral blood cells [47]. If the AP site damage observed in the present study was dependent upon an interaction with this enzyme, we should have also observed this kind of damage in PHA-stimulated whole blood cells. However our findings were not consistent with this prediction.

It is our view that the induction of damage at DNA-AP sites observed in non-stimulated whole blood cells herein was due to interaction of the drug metabolites with cellular targets beyond topoisomerase IIβ (which was not present at high levels), such as DNA and/or proteins. Interaction of the drug metabolites with these alternative targets can cause DNA damage by the generation of free radicals [28]. Another possible explanation could be that DNA repair status in non-stimulated whole blood cells are less active than stimulated whole blood cells to remove this kind of DNA damage [48]. Moreover, the reduced DNA damage in the presence of the antioxidant AA suggests that etoposide-hydroquinone phenoxyl radical is the responsible mediator of these effects [27,49].

## Conclusion

In summary, our data show that etoposide can produce differential forms of DNA damage in PHA-stimulated and non-stimulated blood cells. These results could have important implications for elucidating the mechanisms associated with the development of secondary malignancies (principally acute mielocitic leukemia) that are associated with the use of etoposide as an antineoplastic drug [50].

## Competing interests

The authors declare that they have no competing interests.

## Authors' contributions

ER was the project leader and directed the study; PM participated in data acquisition and generated experimental data. ET performs the statistical analysis of the data. MV was the main investigator of the study, performed the experimental design and the manuscript draft. She is the corresponding author. All authors reviewed and approved the final manuscript.

## Pre-publication history

The pre-publication history for this paper can be accessed here:

http://www.biomedcentral.com/1471-2407/9/398/prepub
